# A qualitative evaluation of the implementation of guidelines and a support tool for asthma management in primary care

**DOI:** 10.1186/s40733-016-0023-9

**Published:** 2016-05-04

**Authors:** Kim Watkins, Colleen Fisher, Jila Misaghian, Carl R. Schneider, Rhonda Clifford

**Affiliations:** 1 School of Medicine and Pharmacology, The University of Western Australia, Perth, Australia; 2 School of Population Health, The University of Western Australia, Perth, Australia; 3 Faculty of Pharmacy, The University of Sydney, Sydney, Australia; 4 Pharmacy Program, School of Medicine and Pharmacology, The University of Western Australia, M315, 35 Stirling Highway, Crawley, WA 6009 Australia

**Keywords:** Focus groups, Asthma, Guidelines, Resources, Stakeholders, Community pharmacy

## Abstract

**Background:**

Asthma management in Australia is suboptimal. The “Guidelines for provision of a *Pharmacist Only* medicine: short acting beta agonists” (SABA guidelines) and a novel West Australian “Asthma Action Plan card” (AAP card) were concurrently developed to improve asthma management. The aim of this qualitative research was to evaluate the collaborative, multidisciplinary and multifaceted implementation of these asthma resources and identify the lessons learnt to inform future initiatives.

**Methods:**

Feedback was sought about the implementation of the SABA guidelines and the AAP card using focus groups with key stakeholders including pharmacists (×2), pharmacy assistants, asthma educators, general practitioners, practice nurses and people with asthma (patients). Audio recordings were transcribed verbatim. Data were analysed thematically using constant comparison. The common themes identified from the focus groups were categorised according to a taxonomy of barriers including barriers related to knowledge, attitudes and behaviour.

**Results:**

Seven focus group sessions were held with 57 participants. Knowledge barriers were identified included a lack of awareness and lack of familiarity of the resources. There was a significant lack of awareness of the AAP card where passive implementation methods had been utilised. Pharmacists had good awareness of the SABA guidelines but pharmacy assistants were unaware of the guidelines despite significant involvement in the sale of SABAs. Environmental barriers included time and workflow issues and the role of the pharmacy assistant in the organisation workflows of the pharmacy. The attitudes and behaviours of health professionals and patients with asthma were discordant and this undermined optimal asthma management. Suggestions to improve asthma management included the use of legislation, the use of electronic resources integrated into workflows and training pharmacists or practice nurses to provide patients with written asthma action plans.

**Conclusions:**

Greater consideration needs to be given to implementation of resources to improve awareness and overcome barriers to utilisation. Attitudes and behaviours of both health professionals and patients with asthma need to be addressed. Interventions directed toward health professionals should focus on skills needs related to achieving improved communication and patient behaviour change.

**Electronic supplementary material:**

The online version of this article (doi:10.1186/s40733-016-0023-9) contains supplementary material, which is available to authorized users.

## Background

Asthma remains a significant health problem in Australia and is associated with significant morbidity, mortality and decreased quality of life [1, 2]. Patients with asthma are required to make day-to-day decisions about how to manage their health. Effective self-management requires the collaboration of all the members of the primary health care team, including general practitioners, community pharmacists, asthma educators and practice nurses, in collaboration with the patient. Effective self-management should also involve the use of a written asthma action plan and appropriate use of “preventer” and “reliever” medications [1]. Written asthma action plans have been proven to reduce mortality, hospitalisations and urgent GP visits [1]. Regular use of “preventer” medications, in appropriate patients, controls the disease and prevents exacerbations [3]. The drug class that is primarily used for this purpose is inhaled corticosteroids. Asthma “reliever” medications are short-acting bronchodilator medicines that provide rapid symptom relief. However it is also acknowledged that regular or excessive reliance on these medications can contribute to poor asthma control and can put the patient at risk of a severe, possibly life-threatening, exacerbation of the disease [1, 4].

Despite the availability of effective evidence-based management strategies, asthma management in Australia remains suboptimal. Ownership of written asthma action plans remains low, at under 25 % nationally, even though they have been recommended in national guidelines for more than 20 years [1, 3, 4]. Analysis of dispensing data indicates that most inhaled corticosteroids are neither prescribed nor used according to current asthma guidelines and there is an over-reliance on “reliever” medications [1].

**Fig. 1:**
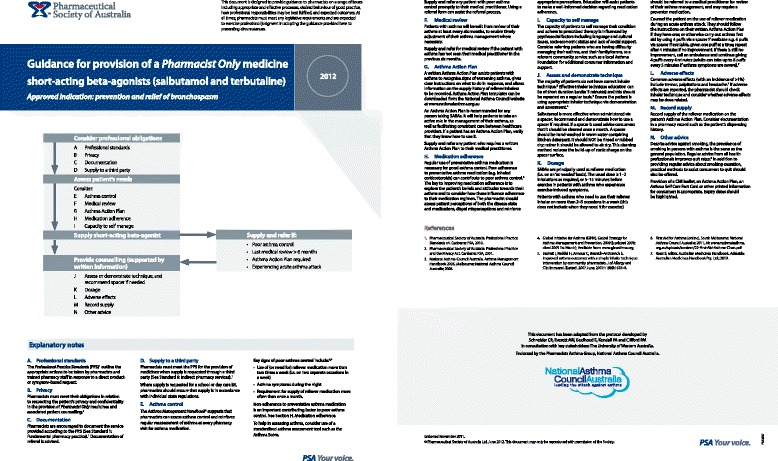
SABA guidelines in PDF format

**Fig. 2:**
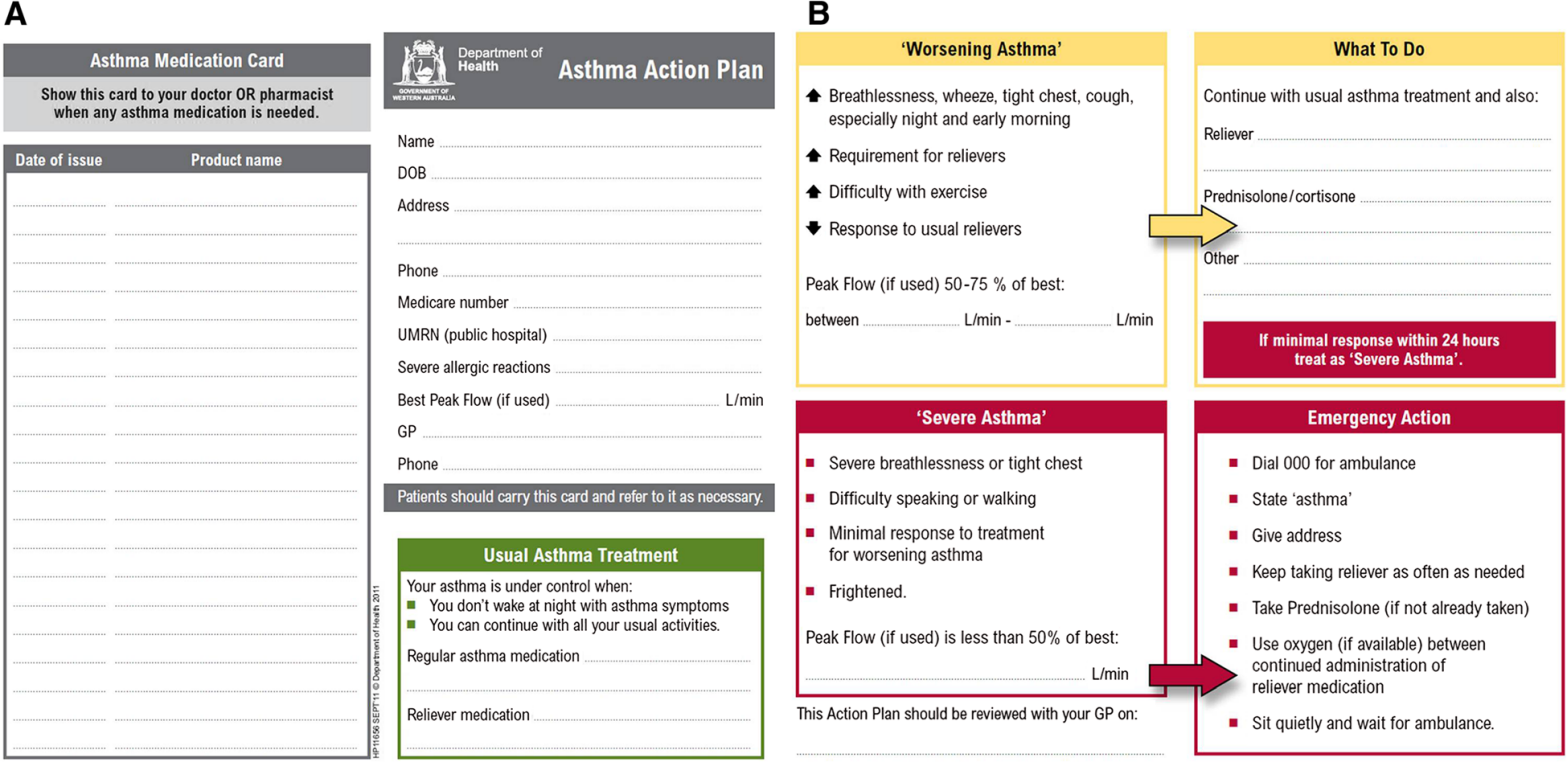
**a** Asthma action plan card (front) in JPEG format. **b** Asthma Action Plan Card (back) in JPEG format

In Australia, community pharmacists are the most frequently accessed primary health care provider [5]. They also play a key role by supplying the medications used to treat asthma. This responsibility is even more critical due to legislation that allows patients to access “reliever” medications without a prescription from their general practitioner (Schedule 3–“*Pharmacist Only Medication*”) [6]. The sale of reliever medications must be under the direct supervision of a pharmacist, which means, pharmacists may often be the only health care professional in a position to regularly assess the patient with asthma. Despite the importance of this role, previous research demonstrated patient assessment, in non-prescription asthma reliever purchases in community pharmacy in Western Australia, to be inadequate [7]. Subsequently the “Guidelines for provision of a *Pharmacist Only* medicine: short acting beta agonists” (SABA guidelines) (Fig 1) were developed to outline best practice for pharmacists. In 2011 the Pharmaceutical Society of Australia (PSA) adopted the SABA guidelines nationally [8]. Concurrently the Health Department of Western Australia’s Respiratory Health Network developed another novel multidisciplinary resource, the Asthma Action Plan (AAP) card (Figs. 2a, b). The AAP card is a portable credit card sized tool that contains a written asthma action plan and includes a section to record medication purchases. It was designed to complement the guidelines, streamline the referral process, encourage patient self-management, improve collaboration and communication and increase the ownership of written asthma action plans [9, 10].

The implementation of the SABA guidelines and The AAP card, was undertaken by the University of Western Australia (UWA), the Respiratory Health Network of the Health Department of Western Australia (HDWA), the Asthma Foundation of Western Australia (AFWA) and the Pharmaceutical Society of Western Australia (PSWA) [10]. The intervention was unique because of its collaborative, multifaceted and multidisciplinary approach. The multifaceted implementation involved targeting pharmacists, patients and general practitioners and used strategies including lectures, educational outreach (academic detailing), educational information packs and media releases via professional networks [10]. The 4-month implementation resulted in the distribution of more than 47,000 AAP cards and provision of academic detailing and/or information packs to more than 500 pharmacies (including all pharmacies in the Perth metropolitan area) [10].

The aim of this research was to evaluate the implementation of the “Guidelines for provision of a *Pharmacist Only* medicine: short acting beta agonists (SABA guidelines)” and “Asthma Action Plan (AAP) card”. The specific objective was to understand the successes and failures of this initiative in influencing health professional practice and patient asthma management to inform future resource implementation.

## Methods

### Ethics approval

Ethics approval was obtained from the UWA Human Research and Ethics Committee (HREC RA/4/1/5000). In accordance with the approval requirements written informed consent was obtained from all participants in this research.

### PICOT framework for initial implementation

Table 1 outlines the details of the initial implementation of the asthma resources using the PICOT framework.

**Table 1:** PICOT framework for initial implementation [10]

PICOT	Prompts
Population	Stakeholders in asthma management in the primary care setting including:
	Pharmacists, pharmacy assistants, asthma educators, general practitioners, practice nurses and people with asthma.
Intervention	Collaborative, multifaceted and multidisciplinary implementation of AAP card and national endorsement and implementation of the SABA guidelines including a comprehensive educational outreach programme.
Comparison	Usual practice.
Outcomes	Increased ownership and use of written asthma action plans by patients and SABA guideline based practice by pharmacists.
Timing	Intervention carried out over a 4-month period between November 2010 and February 2011

### Evaluation methods

A qualitative approach underpinned epistemologically by pragmatism and utilising focus groups, was used for this research [11, 12]. The advantage of a qualitative approach is that it provides an understanding of the perspective of stakeholders and the barriers that exist to practice change [13]. Implementation research has demonstrated that identification of barriers and tailoring implementation strategies to overcome barriers, may lead to improved patient care [14].

Seven focus group sessions were held with key stakeholders in asthma management in the primary care setting and those targeted in the SABA guideline and AAP card implementation. Including a range of stakeholder groups from care pathways facilitated a greater understanding of the barriers resulting from a lack of collaboration and barriers associated with perceptions of individuals about their role. As such, focus groups were conducted separately with pharmacists (×2), pharmacy assistants, asthma educators, general practitioners, practice nurses and people with asthma (patients). Having groups of participants with similarities allowed for exploration of shared experiences to gain an understanding of the issues around asthma management. This approach is consistent with the views of Krueger and Casey who noted there was a decrease in the quality of data from groups composed of highly diverse participants [15].

### Participant recruitment

Purposeful sampling was used to recruit focus group participants [16]. Initial recruitment was via professional organisations and patient networks. Additional methods included the use of letters and direct emailing of individual pharmacies, medical practices and contacts from the primary author’s professional network. Strategies were triangulated where necessary to ensure adequate recruitment numbers. The aim was to recruit between four and 12 participants as is consistent with optimal idea exchange within focus groups [15, 17, 18]. Written informed consent was obtained for participation and for audio recording. Participants from all groups received a retail gift voucher of nominal value to compensate for their time and travel costs, except for the asthma educators who attended the session as part of their normal working hours.

### Focus group format

The quality of data generated from focus groups depends on the skill and impartiality of the facilitator [11, 15]. An experienced focus group facilitator, who was not a stakeholder, was recruited from the School of Population Health at the University of Western Australia to facilitate discussions. She was subsequently invited to collaborate with the research team as a co-author. A researcher (KW) and the facilitator (CF) attended each focus group session. The researcher did not participate in discussions but took detailed field notes of the session. Krueger noted that it was important for the person responsible for analysis to be present in focus groups due to the subtleties of mood, energy and enthusiasm that convey rich information that cannot be determined via transcripts alone [15]. “Immersion” in the data provided a deeper understanding and enhanced interpretation [13, 15]. Engaging the same facilitator allowed for minimal variation in delivery style between focus groups. The facilitator was provided with a structured format to ensure uniformity in the way focus groups were conducted. This included: An introduction, explanation of the ground rules (e.g. manners, confidentiality), explanation of procedural issues (e.g. audio recording) and information about participation and consent. This structured approach provided reassurance to participants that they were in a safe and non-threatening environment [16]. The focus groups were timed to last for approximately 1 hour. The priority in holding focus groups was to get a broad perspective from a variety of stakeholders. While there was stakeholder heterogeneity between groups repetition of common themes was evident providing confidence that saturation of the main themes was achieved [12, 15].

As the SABA guidelines are clinical guidelines specifically for pharmacists, only pharmacy staff (pharmacists & assistants) discussed the guidelines in focus group sessions. The AAP card is a multidisciplinary resource and as such was discussed in all of the focus group sessions. Where participants were unaware of the card they were given a sample of the resource along with a brief explanation of its purpose and asked to speak hypothetically about their impressions of the tool.

Topics for discussion included participants’ knowledge of the guidelines and card, opinions about usefulness and usability of the resources, barriers inhibiting their use and ideas to improve the resource and/or asthma management (Additional file 1).

### Data collection

Data were collected in three ways from the focus group sessions:

1. Researcher observations and field notes
2. Audio recordings
3. Participant demographics and written summary of key opinions.

Field notes, which have been argued to play an important role in accurately representing discussions [11] included detailed participant responses, descriptive information and numerical data about resource awareness, as has been described by Krueger and Casey [15]. The field notes were utilised in the analysis in conjunction with the transcripts and provided context and emotion not always conveyed by the written quotes alone.

Participants were asked at the end of the focus group discussion to independently fill out a summary sheet of perspectives. Summarising critical points in this way was a method of confirming the accuracy of findings (Additional file 2).

### Data analysis

Having the primary researcher (KW) present at all focus group sessions provided opportunities for the analytical process to begin during data collection [15, 19]. Audio recordings were transcribed verbatim. To ensure methodological rigour, transcripts were thematically analysed independently by two researchers (KW, JM) [11, 15]. Inter-coder agreement was checked and a moderator was available (CS) where necessary to achieve consensus [12, 13]. The analysis included inductive category development to allow the categories to be informed by the data rather than using a pre-conceived framework approach [13, 16]. The process of analysis involved:

- Step 1: Immersion in the data through reading and re-reading transcripts
- Step 2: Highlighting words or phrases that capture key thoughts and making notes of first impressions on analysis
- Step 3: Labelling similar thoughts with a code (development of coding scheme)
- Step 4: Codes grouped into broader categories based on similarities
- Step 5: Themes identified based on a greater understanding of the relationships between categories and the identification of patterns in the data.

The technique used was consistent with constant comparison [11, 15, 19]. This process was reflective and iterative and involved continual refinement and revision of codes and broader categories over the course of the analytical process. It also involved concurrent development of an understanding of the relationships between codes and categories through not only the use of transcripts but also field notes. This conceptual understanding led to the identification of themes from the data.

Thematic analysis involved comparison of similarities and differences across stakeholder groups [15]. It also provided clarity about the barriers and facilitators to the use of the resources and asthma management in general. An inductive approach was taken with the thematic analysis and subsequent application of the knowledge, attitudes and behaviour theoretical framework and taxonomy allowed for interpretation of what influences guideline-based practice [20, 21]. Briefly, the taxonomy comprises 7 general categories relating to knowledge (lack of awareness or lack of familiarity), attitudes (lack of agreement, lack of self-efficacy, lack of outcome expectancy, or the inertia of previous practice), and behavior (external barriers) [20]. Use of a taxonomy allows for greater applicability of the results of this research.

Summary sheet data was analysed in conjunction with the focus group transcripts.

## Results

### Focus group participation and demographics

A total of seven focus group sessions were held with 57 participants. Nine and ten participants attended the two pharmacist focus groups respectively. Other groups included: pharmacy assistants (11 participants), practice nurses (six participants), asthma educators (five participants), general practitioners (six participants) and patients (ten participants). The patient group had an even gender spread (four male and six female) and an age range of 21 to 80 years with a mean age of 51.8 years. The participant demographics are shown in Table 2.

**Table 2:** Focus group demographics & knowledge of the asthma resources

Focus group participants							
	Pharmacist group 1	Pharmacist group 2	Pharmacy assistant group	Practice nurse group	Asthma educator group	General practitioner group	Patients with asthma (patient) group
Participant numbers Total (male:female)	9 (3:6)	10 (0:10)	11 (1:10)	6 (0:6)	5 (0:5)	6 (2:4)	10 (4:6)
Age (Average) (Range)	41.5 (26–57)	31 (23–60)	27.6 (19–49)	61.7 (50–72)	42.6 (23–60)	49 (41–57)	51.8 (21–80)
Hours of work (Full time)	3	9	6	0	5	3	N/A
Awareness of Asthma Action Plan card	8 (89 %)	10 (100 %)	10 (91 %)	2 (33 %)	5 (100 %)	1 (17 %)	2 (20 %)
Use of Asthma Action Plan card (current or previous)	5 (56 %)	5 (50 %)	5 (45 %)	0 (0 %)	2 (40 %)	0 (0 %)	0 (0 %)
Awareness of SABA guidelines	8 (89 %)	10 (100 %)	0 (0 %)	0 (0 %)	5 (100 %)	0 (0 %)	N/A

### Benefits of resources

Community pharmacists displayed a positive attitude towards the SABA guidelines. In this study they articulated clear benefits in having formal clinical guidelines for the non-prescription supply of short acting beta agonists. They saw the guidelines as a useful education, clinical and communication resource that enhanced professionalism.

*I have been using them [the guidelines] as a teaching technique for new staff, students, more as a training guide than anything. (PHARMACIST)*

*It’s a good starting point and you might just tease out something from them [patients] by working through the guidelines…[something] they hadn’t been doing right. (PHARMACIST)*

*It’s a way of getting information from patients and finding if things are working as they’re supposed to be. (PHARMACIST)*

*If we’re trying to project an image of being professional rather than competing with supermarkets then it is a good idea for all pharmacies to adopt. (PHARMACIST)*

There was also recognition that having formal guidelines for non-prescription supply of SABAs improved medication accessibility for patients.

*Without these sort of guidelines it is not likely a lot of these medicines would be available over the counter…It’s probably reducing the workload on doctors as well. (PHARMACIST)*

While all participants in the groups could hypothetically acknowledge the potential benefits of the AAP card, the focus and enthusiasm for discussion related to negative aspects of the AAP card. Many of the barriers mentioned challenged the realisation of the hypothetical benefits in practical application. Furthermore barriers often related to development and use of written asthma action plans and asthma management in general rather than specifically the AAP card.

### Barriers to resource use

**Fig. 3:**
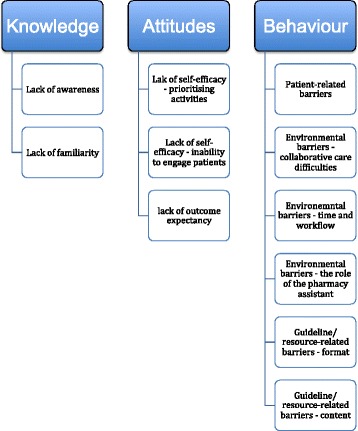
Barriers to use of asthma resources

The barriers identified to the use of the SABA guidelines and AAP card are shown in Fig. 3 *.* The barriers were classified according to an existing taxonomy [20].

### Knowledge barriers

#### Lack of awareness

There was high awareness of the SABA guidelines among pharmacists (group 1 89 %, group 2 100 %) and similarly a high awareness of the AAP card (group 1 89 %, group 2 100 %). In contrast the participants in the pharmacy assistant group were completely unaware of the SABA guidelines (0 %) but had a good awareness of the AAP card (91 %). The only other group with participants who had much familiarity of the asthma resources was the asthma educator focus group. All of the asthma educator participants (100 %) were aware of both the SABA guidelines and the AAP card. Participants in the general practitioner and practice nurse group were completely unaware of the SABA guidelines and had minimal awareness of the AAP card (33 and 17 % respectively). Only 20 % of patient group participants were aware of the AAP card, despite the card being marketed as a patient resource.

#### Lack of familiarity

Although the majority of pharmacists, pharmacy assistants and asthma educators were aware of the AAP card only 49 % had previously used the cards in practice and no participants had continued to use them. No patient reported using the AAP card to assist in self-management of his or her asthma. This lack of experience with use of the AAP card meant that in many instances the focus group participants were speaking about initial impressions of the tool and its hypothetical use. As a consequence our focus changed and the discussion broadened towards a more general conversation about issues in asthma management and written asthma action plans.

### Attitudinal barriers

#### Lack of self-efficacy–the need to prioritise work

Pharmacists mentioned that with the time constraints they often faced in daily practice that they had to prioritise their work. They felt that they couldn’t do all the tasks for ideal patient management.

*Sometimes in the pharmacy there’s usually only a single pharmacist on duty…and you have to run…. that’s part of working, isn’t it, sometimes you just keep on doing what’s most important, and it’s time. (PHARMACIST)*

#### Lack of self-efficacy–inability to engage patients

While patient behaviors were considered to be obstructive to guidelines-based care there was also evidence that the attitude of pharmacists was that there was little they could do to alter the situation. There was a feeling that they were disempowered to practice according to the SABA guidelines.

*The main problem with the guidelines is that the people who need the discussion to happen the most are the ones who are least likely to talk to you in a lot of instances. (PHARMACIST)*

*So they [the patients] seem to think it is their god-given right to be handed their Ventolin® and just resent being asked questions. (PHARMACIST)*

#### Lack of outcome expectancy

When it came to the discussion about the AAP card the views of pharmacists and pharmacy assistants were particularly important, as they were essentially the only group that included participants that had attempted to use the resource.

Pharmacists felt unsupported in their attempts to use the card. This included lack of stakeholder support from patients, general practitioners and even the health system (in terms of legislative support to encourage better self-management by patients with asthma). All pharmacists indicated they had “given” up using the card.

*If [the patients] are not interested …I [the pharmacist] am not. (PHARMACIST)*

*I still give [the card] out, and everyone in my shop still gives it out, but we never see, we rarely see anyone bringing it in a second time.* *(PHARMACY ASSISTANT)*

Pharmacy assistants had a negative view of patients with asthma and little understanding of the reasoning behind their behaviour and resistance to engage.

They were also more likely than pharmacists to view the AAP card as a punitive monitoring tool than as a referral tool or a way of improving asthma action plan ownership.

*If customers are using these [cards] you know they are genuine. You don’t have to be suspicious of them just coming in and buying reliever medications. (PHARMACY ASSISTANT)*

Pharmacists were more likely to see the reluctance for patients to engage in conversation as a result of lack of understanding, however, pharmacy assistants tended to focus on negative perceived patient attributes, such as laziness.

*Vagueness…people might start off with [the card] the first time and then they can’t be bothered. (PHARMACY ASSISTANT)*

*Waste of time, as people get too lazy to bring in card*

*(PHARMACY ASSISTANT- Summary Sheet)*

While there was much malignment of patient unwillingness to engage, it would seem that attitudes of pharmacists and pharmacy assistants in the community pharmacy were not conducive to improving the situation. Although these attitudes were conveyed in relation to the resources, it was clear that they applied to engaging with asthma patients in all instances. Not just in specific circumstances.

### Behavioural barriers

#### Patient-related barriers

There was much discussion surrounding resistance of patients to interactions with health professionals and the view that patients preferred to self-manage their asthma. Patient behaviour and engagement difficulties were seen as a significant barrier to the success of the card and also as a barrier to optimal management of asthma. Health professionals believed that patients with asthma did not want their help and would “do their own thing” regardless of the advice they were given. Some found patients became aggressive in response to attempts to intervene. There was little acknowledgement of the patient perspective or reflective thinking about the motivations behind patient behaviour.

*People get, even with the pharmacist, really antsy. [They say] I’ve been using this for a while, why do you need to ask me all these questions. (PHARMACIST)*

*Since they are the customer and they are going to get [the reliever medication] anyway they just go…yeah, yeah, yeah, yeah, yeah [to your questions]. (PHARMACY ASSISTANT)*

*I did try once to get people to come back for the asthma education plan and come the next visit, nope, it’s really dispiriting. (PRACTICE NURSE)*

*Patient unwilling–capability (PRACTICE NURSE–Summary Sheet)*

*I’ve never had [a card] handed over to me to fill out…. I’ve never had a client bring one in…never. [Chorus of agreement] (ASTHMA EDUCATOR)*

*The majority of asthmatics I see rely purely on Ventolin® or similar, and the reason’s because the preventers are cortisone based and there’s enormous prejudice against them. They won’t use them, particularly parents. (GENERAL PRACTITIONER)*

Even the patient group acknowledged that they themselves were often ambivalent and “part of the problem” in achieving optimal asthma management.

*I wouldn’t be bothered, couldn’t be bothered carrying [the card] around…You’d have to be just about dying not to be able to tell somebody to dial 000, you’ve got asthma. (PATIENT)*

*It’s probably only a matter of time before I have another severe attack. But at this stage, if it’s not happening, you don’t think about it. (PATIENT)*

*I’d been wheezing all day and hadn’t paid attention to it [my asthma]…my husband’s a GP but he was out and by the time he came home then I passed out and he had to resuscitate me. (PATIENT)*

#### Environmental barriers–collaborative care difficulties

Although patient engagement was considered the major barrier to use of written asthma action plans (including the AAP card) and optimal asthma management, the general practitioner was also seen by other stakeholder groups to be unsupportive of collaborative care initiatives. There was frustration in the fact that general practitioners had the ultimate responsibility to provide patients a written asthma action plan and were seen not to be fulfilling that role.

*At the end of the day [written asthma action plans] have to be done by a GP. We can’t give this information…we can refer…. I don’t think GPs are being proactive enough in making sure patients actually have one. (PHARMACIST)*

*Doctors just don’t seem to follow up and do it [fill in the card with an action plan]…. Patients come back and say their doctor didn’t want to know about it. (PHARMACIST)*

*I’ll sometimes write it out [a written asthma action plan] and the doctor will sign it…but it’s still up to them actually…that’s the biggest problem. (PRACTICE NURSE)*

*It is just not going to happen [GPs writing asthma action plans]. I think GPs are as complacent about asthma as the general community.* *(ASTHMA EDUCATOR)*

The attitudes of many health professionals showed little understanding of the GP perspective and the difficulties they faced in managing patients with asthma.

General practitioners acknowledged that there were barriers in their practice that prevented them from managing patients with asthma and providing patients with written asthma action plans. They particularly felt frustrated that patients would only present when unwell, which did not allow for chronic disease management, only the provision of acute care.

*People will present when they get a flare up, get some treatment, but they don’t come back to discuss a plan for next time.* *(GENERAL PRACTITIONER)*

*You’re just dealing with the acute flare up and managing that…They don’t really want to come in afterwards and do…longer term planning when they’re well, because they’re well and they don’t feel like attending then, to do that, they’ve got too many other things to do. (GENERAL PRACTITIONER)*

*It’s usually just; oh they need a script along with all the other stuff. They don’t always have the time to deal with asthma…and it’s down the bottom of their list. (GENERAL PRACTITIONER)*

*Time in general practice (GENERAL PRACTITIONER- Summary Sheet)*

Practice nurses echoed this sentiment.

*You give them opportunistic education…not a formal clinic thing, because it’s just impossible. (PRACTICE NURSE)*

*We do really get more acute people…they arrive to us, having an acute asthma attack. (PRACTICE NURSE)*

*The asthma two-step plan…we’re not really doing that. (PRACTICE NURSE)*

#### Environmental-related barriers–time and workflow

Time barriers were another issue identified by health professionals and pharmacy assistants as hampering their ability to use the card and effectively support optimal asthma management.

*Even in the quietest pharmacies you’re always battling for time. Either you’re in a rush or they’re in a rush…so there is a time factor. (PHARMACIST)*

*There’s a time factor as well. They [patients] are going to be too hostile, and they don’t want to stand around waiting [for us to fill in the card]. (PHARMACY ASSISTANT)*

*I don’t have time to sit down and really do [education]…how you’re supposed to…then get them back later [for a written asthma action plan].*

*(PRACTICE NURSE)*

*You’ve got to actually fit in the spirometry checks as well…you’ve got to work out if you’ve got time…so it just gets a bit difficult to provide longer term management advice to patients. (GENERAL PRACTITIONER)*

*Time consuming–should be more faster process so that customer would be inclined to use (the AAP card). (PHARMACY ASSISTANT- Summary Sheet)*

#### Environmental-related barriers–the role of the pharmacy assistant

Of particular interest in the pharmacy assistant focus group discussion was the description of their role in SABA sales. Currently the scheduling of salbutamol and terbutaline inhaler medications (SABAs) in Western Australia is according to the Standard for the Uniform Scheduling of Medicines and Poisons (SUSMP) [22]. This recommends a classification for these medications as Schedule 3 or “Pharmacist Only” medications. The West Australian Poisons Regulations of 1965 indicate that, “a Schedule 3 substance should only be sold by way of direct personal sale by a pharmacist or an intern pharmacist under the direct personal supervision of a pharmacist” [6]. They also state that, “the pharmacist must take all reasonable steps to ensure there is a therapeutic need for the product” [6]. Despite the legislative requirements for pharmacist involvement and a universal lack of knowledge of the SABA guidelines, pharmacy assistants indicated that they were highly involved in the provision of SABAs. This involvement represents an environmental barrier to optimal asthma management.

#### Guideline/resource-related barriers–the format of the Asthma Action Plan card

There was a great deal of discussion surrounding the format of the card. Most stakeholders commented that patients had overloaded wallets and were not likely to carry around a card that didn’t really serve a purpose from the patient’s perspective.

*I think people think it’s too bulky…they don’t want to carry it around in their purse. (PHARMACY ASSISTANT)*

*The customer is not going to carry this around purely to record when they buy their Ventolin®. That’s really the issue. They don’t see any value in that. (PHARMACIST)*

*Bulky/cumbersome: could get tatty (ASTHMA EDUCATOR–Summary Sheet)*

There was criticism of the paper-based format when most health records are being converted to an electronic format.

*A card of this size is just another barrier and so much of our recording and things we do is electronic. I think it has become almost a little bit old fashioned. (PHARMACIST)*

*The recording of Ventolin® is something that either has to be electronic or it’s not going to happen. (PHARMACIST)*

*I think all of us would agree that [a computer based template] would be the format we’re likely to use, more than a card, because we’re not filling out a card. (ASTHMA EDUCATOR)*

The paper-based format was also seen as problematic due to its lack of durability.

*I think guys put it [the AAP card] in their back pockets… and it just gets really tattered. (PHARMACIST)*

#### Guideline/resource-related barriers–the content of the Asthma Action Plan card

There was also criticism of the content of the card. Pharmacists particularly felt the dual purpose of a written asthma action plan and beta-agonist record was confusing.

*Do we want to highlight that they need an asthma management plan, get them to a GP? Or do we actually want a mechanism for recording? …We’re trying to do two things here at cross-purposes. (PHARMACIST)*

Many indicated that the language of the card was inappropriate for patients.

*I’d like to see the wording re-done so it’s more patient friendly.* *(PRACTICE NURSE)*

*[The card] is not really culturally appropriate for the indigenous population…well probably a number of groups…English as a second language. (ASTHMA EDUCATOR)*

It was felt that important information such as asthma first aid and definition of beta-agonist over-use was missing.

*[The card contains] emergency action but it is not really first aid. It wouldn’t be useful…. (PRACTICE NURSE)*

*I don’t think that [the card] would get the message across that you should only be using one (Ventolin®) a month. (PATIENT)*

*There’s got to be some sort of awareness that there is a problem if you’re using [reliever medications] “x” amount of times per week…basically our guidelines need to be exploded out to everyone else, so that way everyone is aware…I’m using [my medication] five times a week so I’d better go and talk to my doctor, or talk to my pharmacist. (PHARMACIST)*

Information such as peak flow and oxygen use was seen as irrelevant for a generalised tool.

*I mean, worsening asthma, peak flow 50 to 75 per cent. Hardly anyone does peak flow now. (PRACTICE NURSE)*

*Peak flow used to be popular 15 years ago but…. now we never use it…It seems to have died a death. I occasionally use peak flow but it’s not really helpful. (GENERAL PRACTITIONER)*

*When I have given [the cards] out, people have gone to the emergency part and [said] oxygen, so where do I get that? You could read it as; will I need to have oxygen at home? (ASTHMA EDUCATOR)*

Discussions in the asthma educator and general practitioner groups particularly focused on suggesting specific and practical ideas to improve the card content, that addressed many of the issues mentioned above, which they found unacceptable.

*Inadequate detail on front page regarding when asthma is under control (GENERAL PRACTITIONER–Summary Sheet)*

*Instructions to patients need to be clearer*

*(PRACTICE NURSE- Summary Sheet)*

These issues with format and content all constitute a barrier to resource utilisation.

### Solutions for improvements

There were a variety of solutions offered by all stakeholders in all focus groups regarding the SABA guidelines, the AAP card and asthma management in general. However, most of the discussion about improvements in all groups veered towards three main themes: mandatory recording of beta-agonist purchases, development of electronic resources to improve asthma management and using pharmacists and practice nurses to develop written asthma action plans.

#### Mandating of SABA recording

Interestingly there was much interest by pharmacists in strengthening the legislation around non-prescription supply of beta agonists. The view of pharmacists in the group was that mandating the recording of beta-agonists would assist patient engagement and SABA guideline compliance.

*If it’s mandated, and every pharmacy has to do it [recording patient details], then they [the patient] can’t skip a pharmacy and go down the road where nobody asks questions because it’s mandated and they are going to get asked wherever they go. And it forces…forces them to engage and forces them to actually have a conversation with the pharmacist. (PHARMACIST)*

*That [mandating] would be a good way of changing expectations…It’s not that they [patients] don’t have time, it’s that they expect when they go into a retail shop that they’re not going to be asked questions. That they come in get what they want in a second and walk out. (PHARMACIST)*

*I think the only way that it [the guidelines] is actually going to produce dramatically improved outcomes is if it’s mandated. (PHARMACIST)*

*People could sign a consent form [when purchasing asthma relievers] …they could get phone calls from education nurses and information sent out… stuff like that. You could record [asthma relievers], if they did that…it would actually help. (PHARMACIST)*

The fact that the AAP card included a beta-agonist recording function was seen as pointless without legislation enforcing the recoding function.

*It’s not mandatory so you just sort of lose momentum. (PHARMACIST)*

*[Mandatory use of the card] could make my life harder. Well more work for us to do, but at least you know then that they have to use it and they have to record it, because they can’t buy it otherwise. (PHARMACY ASSISTANT)*

Despite the popularity of the notion of mandatory recording of SABA sales there were some concerns raised about the importance of maintaining patient accessibility to medication and the potential unacceptability for patients of legislative change.

*In certain places you have to have an asthma card or have come from the doctor [to obtain a beta-agonist] …there is a danger there, because if you have someone who is having an asthma attack what are you going to do? (PHARMACIST)*

*The backlash [from mandatory recording of reliever medications] would be huge. (ASTHMA EDUCATOR)*

#### Electronic resources

The criticism of the AAP card being a paper-based resource was matched by enthusiasm for the development of electronic resources to improve asthma management.

*I don’t know whether there’s something electronic that could be done these days, an App on the phone or something… So much of our recording and things we do is electronic. (PHARMACIST)*

*Electronic…something that you can scan on a program…something that the [medication] history comes up, how many [reliever medications]…starting it that way. (PHARMACY ASSISTANT)*

*[Electronic action plans] are good because you can see exactly what they’ve had. If you’re just writing one [an action plan] out they [patients] tend to lose it or never bring it back when you have the next appointment.* *(PRACTICE NURSE)*

*I think keeping an electronic record of your preventatives; your Ventolin® and so forth would be valuable in this day and age. (PATIENT)*

However there was also recognition that electronic formats would not necessarily suit all demographics.

*Not everybody has computer, especially older patients.* *(PHARMACY ASSISTANT)*

*I think a lot of my patients…depends on age…80 year olds wouldn’t use electronics. (GENERAL PRACTITIONER)*

Overall most health professionals felt disempowered to intervene in asthma patient’s inappropriate self-management and reliance on asthma reliever medications and felt that the card did not offer solutions to this issue with its imperfect paper-based recording function.

#### Improving written asthma action plan ownership

In terms of addressing the issue of lack of patient ownership of written asthma action plans, again the card was not seen as the solution. Asthma educators particularly felt that currently existing written asthma action plan templates were superior to the card.

*You only have to look at [these other plans] and you see, they look a lot more colourful and maybe more likely to stick in people’s minds I think.* *(ASTHMA EDUCATOR)*

*When explaining asthma action plans to clients, I find these [other plans are] really simple…they’re all on one page to look at whereas [with the AAP card] you’re flipping. (ASTHMA EDUCATOR)*

Pharmacists felt that they could play a role in writing plans for patients.

*What would be the consequences if we [pharmacists] did actually set out an action plan? The doctors would get upset but the patients would get one. (PHARMACIST)*

*We could do an asthma educator course and become the person who does the plan instead. (PHARMACIST)*

However there was also discussion about the need to up-skill pharmacists to take on the role of preparing written asthma action plans for patients.

*I think pharmacists do need up-skilling though to provide this service. [Written asthma action plans] (PHARMACIST)*

Additionally there was some concern about the need to address time and remuneration barriers before pharmacists could undertake this expanded clinical role.

*I think it comes back to the time and remuneration factor, you know that if you are going to employ, or have pharmacists, and have the time to do that [write asthma plans]. There needs to be balance. I think it is a great thing to do and a great service but we need to be able to get the pharmacist out of the dispensary and to do that you obviously need to be getting paid for it. (PHARMACIST)*

Similarly some general practitioners felt that a solution to improve written asthma plan ownership could be utilisation of practice nurses to undertake the task.

*Where I would probably end up using that [AAP card] within the context of my practice would be if they came to see the nurse to actually get the action plan done, because that’s when we put aside a bit of time for them to sit with the nurse and have discussions. (GENERAL PRACTITIONER)*

*Do it all themselves, let [practice nurses] get on with [writing asthma action plan]. (GENERAL PRACTITIONER)*

However some were cautious about this proposition and questioned the capability of practice nurses to undertake the role.

*“I’m not sure [about nurses writing asthma action plan]…we have practice nurses but I don’t know that asthma is their strongest area, I think they’d be happy showing people how to use their inhalers but I’m not sure about going through it all.” (GENERAL PRACTITIONER)*

## Discussion

This research explored the views of stakeholders of asthma resources produced and implemented to improve asthma management. Utilising focus groups allowed for an in-depth understanding of the barriers that undermine successful implementation of resources to achieve practice change and improved patient health outcomes. While this research focussed on specific resources, the information gleaned from these focus groups provides insight into key issues of asthma management and effective use of resources to improve health-professional practice and patient engagement.

The lack of awareness and use of the asthma resources was initially surprising given the comprehensive, multifaceted implementation plan used by the collaborative team which included personnel and resources from a university research team, the government Health Department, a professional pharmacy organisation and an asthma organisation [10]. However it is not unexpected given the evidence in the scientific literature on clinical guideline implementation [23–26]. Despite the seemingly thorough approach essentially all of the implementation activities involved educational interventions using passive dissemination, to most professionals, and educational outreach to pharmacists. The difference in awareness of the asthma resources found between groups may be due to the differing dissemination strategies employed. Educational interventions have been demonstrated to be minimally effective, particularly if they simply involve passive dissemination [23–26]. Educational outreach has been found to be effective in medical settings [25], however, in the community pharmacy setting, Watson and colleagues demonstrated no evidence of practice change using this intervention method [27]. Clearly the first step to successful implementation of guidelines and other resources is to ensure that there is not only widespread knowledge of the resource but also a willingness to incorporate it into practice. More consideration needs to be given to the implementation strategies employed to achieve this and there should be less reliance on passive implementation.

Sustained practice change will not result if too many barriers are encountered during initial attempts of practitioners to use a resource. In the case of the SABA guidelines and the AAP card the barriers encountered were many and not just related to the resources themselves. It was evident that attitudes of all stakeholders were influencing behaviours that were detrimental to optimal asthma management. There was a misalignment of asthma management goals and behavioural expectations between patients and health professionals and this discordance seemed to be central to the issue.

Overwhelmingly the perceived difficulties with patient engagement resulted in pharmacists and pharmacy assistants having a pessimistic view of their ability to influence patients to appropriately self-manage their condition. Asthma educators, general practitioners and practice nurses were equally pessimistic and felt they had limited opportunities to provide chronic disease management and patient education. Apathy to engage was even acknowledged by patients themselves. In many instances patients were unaware of poor asthma control or were resigned to having asthma exacerbations and limitations put on their lives by this chronic condition. Patients only focussed on current symptoms and did not have expectations of interventions by health professionals aimed at chronic disease management. These patient attitudes are consistent with much of the literature in asthma [28, 29]. What is also known from the literature is that illness perceptions determine the way in which asthma patients cope and self-manage this condition [30]. The focus group results convey that effective self-management, with the support of health professionals, cannot be achieved without addressing patient attitudes, beliefs and perceptions about asthma. The extent of the issue warrants a community level intervention directed at the patient. Resources such as the AAP card are unlikely to have an impact without patient acceptance and more widespread consultation should be undertaken in resource development.

Equally health practitioner attitudes require addressing. There was little evidence of a patient-centred focus by health professionals or reflection on changes that could be made to allow for more effective patient engagement. Negative perceptions had reduced the motivation of health professionals. In this study there were indications of lack of self-efficacy and poor impressions of outcome expectancy hampering patient engagement. Motivation to change practice needs consideration in any implementation of guidelines and/or resources. Remuneration opportunities may change prioritisation of activities but ultimately improving outcome expectancy is critical. In order to do this, health professionals require not only motivation but also the skills to achieve outcomes. Capability must be addressed. As health professionals saw patient engagement and patient attitudes as a barrier, any asthma management intervention should tackle their skills needs in this area and not just clinical education needs. Health professionals require advanced communication skills and experience with techniques such as motivational interviewing in order to effectively adjust practice and explore and guide patient perceptions [31].

Environmental barriers were also a key concern. It was not surprising that many of the health practitioners mentioned time and remuneration as barriers to practice [21, 32]. This has been well documented. However the most interesting finding related to how organisational factors, in particular the role of the pharmacy assistants, impacted on practice. What became evident, from the focus groups, was that pharmacy-assistant involvement was a barrier to the provision of guideline-based care in the community pharmacy setting. Pharmacy assistants had no knowledge of the SABA guidelines but articulated being highly involved in the provision of non-prescription asthma reliever medications and in some cases saw it as “their responsibility”. This is consistent with prior research in this region that observed in 47 % of non-prescription sales of beta-agonists there was no obvious involvement of a pharmacist [7]. The participation of pharmacy assistants is despite the current legislative requirements for direct pharmacist involvement and despite the fact they are not qualified or trained to undertake this role. Surprisingly, even though current legislative requirements are not being met, both pharmacists and pharmacy assistants wanted the legislative requirements strengthened to include mandatory recording of non-prescription beta-agonist purchases. While this may seem pointless, given current lack of adherence to legislative requirements, mandatory recording may have an impact because it may change the workflows around the supply of SABAs. Current workflows in community pharmacy dictate that pharmacy assistants lacking formal training are often the first and only point of contact for asthma patients who are often resistant to engagement. Mandatory recording may facilitate improvement in asthma management because it may increase the involvement of pharmacists by moving the interaction into the dispensary for recording purposes. This would be desirable because increased pharmacist involvement has previously been found to result in more appropriate medical referral of asthma patients [33]. Mandatory recording would also provide easier opportunities for auditing legislative compliance by pharmacists. Currently the Schedule 3 legislative requirements are not routinely policed and statutory bodies rarely prosecute breaches [34].

Discussions in focus groups about the AAP card evolved into discussions about written asthma action plans in general. While the AAP card had merit in the hypothetical it was a flawed resource that was not going to be accepted and utilised by stakeholders. The stakeholders noted there were other preferable resources but these resources were also underutilised and not serving the purpose of improving asthma management [1–4].

The sole authority of general practitioners to provide written asthma action plans was seen as a barrier to patient ownership of written asthma action plans. General practitioners lamented the lack of opportunity to manage asthma as a chronic illness. Practice nurses talked about the remuneration pathways for writing asthma action plans. In Australia, a general practitioner is eligible to receive a Service Incentive Payment (SIP) for each Asthma Cycle of Care completed [35]. However a minimum of two asthma related consultations must be completed within 12 months and at least one of the consultations needs to be a review consultation planned at a previous consult [35]. Both the general practitioners and practice nurses noted that this 2-step process was difficult to achieve with patients not returning for their review consultations. The observations of this barrier are interesting given that the Asthma Cycle of Care in Australia has previously been modified from a 3-step process (the Asthma 3+ Visit Plan) in response to feedback from respiratory physicians, general practitioners and patients. It would seem that the simplified 2-step Asthma Cycle of Care has not achieved its aims of ensuring patients with asthma are provided on-going monitoring. Non-attendance at asthma disease management consultations is not an issue unique to Australia. Audit data from the UK on avoidable deaths in asthma indicated that 22 % of patients who died had missed routine asthma appointments in the year before death [36].

Suggestions to address the inability of general practitioners to ensure all patients with asthma have a written asthma action plan included practice nurses writing plans and pharmacists being trained to write asthma action plans. Practice nurses may have fewer time barriers than general practitioners but the fact that patients are is still required to present at a general practice surgery does not address the issue of opportunity. Community pharmacists, alternatively, are highly accessible and as medication experts could possibly undertake this role with some up-skilling. This may be an avenue worth exploring to increase patient asthma action plan ownership, given the on-going difficulties over the last 20 years in addressing the issue. Time barriers are likely to be a problem for pharmacists in undertaking this role, given current time pressures articulated in focus group discussions. Appropriate remuneration would allow for employment of extra staff and may increase pharmacist motivation to engage in clinical service delivery [37].

There was much discussion about the AAP card being a paper-based resource. What became evident is that most stakeholders across all demographics prefer electronic resources. This included patient-based resources in the format of smartphone Apps, along with written asthma action plans and medication records compatible with the software that general practitioners and pharmacists already use. A review by Huckvale and colleagues in 2012 assessed the content of apps designed to assist patient asthma self-management [38]. Of the 103 apps assessed the conclusion was that none provided a combination of reliable, comprehensive information and useful self-management functions [38]. While there are plenty of electronic resources available, more consideration needs to be given to the production of unbiased, high quality, evidence–based and simple-to-use resources that health professionals can confidently recommend to patients to promote self-management Organisational barriers relate to characteristics of the work setting such as staff workload, management issues, financial considerations and structural arrangements that govern workflows [39]. These barriers need to be overcome for resource implementation to successfully achieve its intended outcomes. It makes sense to link resources to existing software used by health professionals rather than using extraneous paper-based resources. Pharmacists, and general practitioners frequently spoke about organisational barriers, such as time pressures, as an issue to optimal practice. Resources that fit into existing workflows (e.g. using existing software such as dispensing software or prescribing software) are likely to be more efficient [40].

### Strengths

A key strength of this research was the triangulation of data sources. Engagement with a range of stakeholders enabled multiple perspectives on the topic. The inclusion of pharmacy assistants, who are not health professionals and do not necessarily receive any formal training in medicines and healthcare, provided rich information about their influence on asthma management. The initial implementation strategy of the SABA guidelines involved pharmacists only and did not acknowledge the workflows in this setting that have pharmacy assistants on the front-line of patient interaction. Running a pharmacy assistant focus group proved valuable in allowing for unconstrained discussion by assistants who may have felt intimidated by a structured interview or in a situation where pharmacists were present [11, 18]. Member checking was undertaken in the form of a written questionnaire.

### Limitations

It is acknowledged that in qualitative research the researchers bring personal values, assumptions and biases to the study. The primary researcher in this study was KW. As a research assistant for the initial SABA guideline collaborative implementation, member of the Health Department of Western Australia’s Respiratory Health Network Executive Advisory Committee and community pharmacist proprietor, KW had a thorough knowledge of the background of the SABA guidelines, AAP card and community pharmacy profession. Awareness of this potential for bias meant that every effort was made to ensure objectivity in the data collection and analysis. This included using a “non-stakeholder” as a moderator, verbatim transcripts of focus group discussions, multi-modal data collection (via field notes, transcripts and summary data) and independent thematic analysis conducted by a second researcher. Member checking did not involve verbal confirmations or access of medical records.

The findings of qualitative research are not generalisable and the ideas generated by these focus group discussions would need quantitative research to assess their applicability [15–17]. Additionally the poor awareness of the resources resulted in many of the discussions being hypothetical, which was an unexpected limitation of the research. Focus groups as a method have inherent limitations and can only explore the barriers and facilitators that participants can articulate. There may be unrecognised barriers and facilitators that were not uncovered by the research methods utilised. While the use of homogeneous focus groups allowed for frank and free discussion it would be useful in future research to attempt focus groups with heterogeneous groups of stakeholders. This would allow for exploration of the issues around lack of collaborative care in asthma, which were evident in the results.

## Conclusions

Using guidelines and resources to improve asthma management requires an effective implementation strategy and consideration of knowledge, attitudinal and behavioural barriers to practice change. The discordant views of patients and health professionals regarding asthma management are a significant barrier to resource implementation and optimal patient health outcomes and this needs to be addressed. Interventions directed toward health professionals should focus on skills needs related to achieving improved communication and patient behaviour change.

Environmental barriers such as workflows need to be understood for effective incorporation of resources into practice.

## Additional files

Additional file 1: Sample facilitator notes. (DOCX 67 kb)
Additional file 2: Patients with asthma at risk: Tools implementation evaluation study. (PDF 84 kb)

## Abbreviations

AAP card: Asthma Action Plan card
AFWA: The Asthma Foundation of Western Australia
HDWA: Health Department of Western Australia
HREC: Human Research and Ethics Committee
PSA: The Pharmaceutical Society of Australia
PSWA: The Pharmaceutical Society of Western Australia
SABA guidelines: Guidelines for provision of a *Pharmacist Only* medicine: short acting beta agonists
SUSMP: Standard for the Uniform Scheduling of Medicines and Poisons
UWA: University of Western Australia
