# Supplementary material for: A qualitative evaluation of the implementation of guidelines and a support tool for asthma management in primary care
Source: Asthma Res Pract. 2016 May 4;2:8. doi: 10.1186/s40733-016-0023-9 (PMC5142429; doi:10.1186/s40733-016-0023-9)
Supplement: Additional file 1: — Sample facilitator notes. (DOCX 67 kb) [file 40733_2016_23_MOESM1_ESM.docx]

**Focus Group Discussion – PARTIES research**

**Asthma Educators and the Asthma Action Plan card**

**A. Introduction**

1. Good [morning, afternoon, or evening], and welcome to our focus group discussion. My name is Colleen Fisher. On behalf of The University of Western Australia, Kim Watkins and myself, I would like to thank you for attending this discussion.

2. This discussion is part of PhD research that is being conducted by The University of Western Australia to investigate opinions about a recently developed resource to aid in asthma management: The Asthma Action Plan card

3. We will begin by going through some house keeping items for today’s discussion:

**B. Ground Rules**

1. There are no right or wrong answers today. We are interested in your awareness of the Asthma Action Plan card and your opinions (from experience or in theory) about the use of this resource. We would like to hear from everyone present, as the more information we get from you, the better our research will be.

2. We encourage everyone to express their opinions, even if you disagree with a fellow participant’s opinion, as we want to have many different points of view. All comments, both positive and negative are welcome. Please remember to be considerate and respectful of everyone’s point of view and give each other the opportunity to speak. It will be much appreciated if everyone speaks one at a time, as this will be easier for us later when we are reviewing the discussion.

**C. Procedure**

1. A tape recorder and scribe will be used during the discussion because we need to pay close attention to what you are saying. After the group discussion, we will listen to the tape to ensure we have correctly transcribed your opinions. The information obtained will be used as part of a thesis. Please remember that you will not be identified in any way.

We will begin the tape recording after our introductions.

2. This discussion is strictly confidential. What you hear and what you say should not be shared with anyone outside this room. This information should stay here. Does everyone agree? Please indicate by putting your hand up.

3. Our session will last about 45 minutes. We will not take a break, but please feel free to get up and use the restroom or help yourself to more food and drink whenever you want.

4. At the end of our discussion, we would kindly ask you to write down your key issues from the topics discussed, along with any other comments you may have, on the sheet provided.

**D. Consent form**

1. Before we begin our discussion, please take a few minutes to complete the consent form in front of you. This session is entirely voluntary and you are able to leave at any time.

**E. Self-Introductions**

1. Let's start by introducing ourselves. As I said before, my name is Colleen Fisher. I am a researcher from the School of Population Health at UWA. I am an accredited focus group facilitator. I will be leading today’s discussion. My colleague is Kim Watkins who is a PhD candidate at UWA undertaking pharmacy practice research. She will be scribing today’s session.

2. Now, please introduce yourselves and let us know a little bit about yourselves such as how long you’ve been an Asthma Educator and where you work. Who would like to start? (Then go clockwise from that person) ……

3. Does anyone have any questions before we begin? The tape will begin recording now.

**F. Discussion**

1. **You have all been provided and Asthma Action Plan card to look at. It is a resource from the Health Department of Western Australia.**
2. **We will start by asking a few simple yes/no questions to everyone in the room:**
3. Have you seen the Asthma Action Plan card before?

Yes ⬜ No ⬜

1. Have you ever used the Asthma Action Plan card?

Yes ⬜ No ⬜

1. Are you aware of the SABA Guidelines for pharmacists for the non-prescription supply of asthma reliever medications?

Yes ⬜ No ⬜

1. **Now I will tell you a little bit about the Asthma Action Plan card:**

- It is designed as a self management tool for people with asthma
- Its aim is to encourage patients to have a written asthma action plan
- Pharmacists can record beta agonist sales on the card

In this discussion you can answer questions from your experience of using the resource OR hypothetically how you think you **could** use the resource.

| **Discussion theme** | **Prompts** |
| --- | --- |
| Describe your role in patient asthma management. | - What is your role in developing written asthma action plans for patients? |
| Describe how you use or could use the Asthma Action Plan card as part of patient management? | - When would you give it out? - Who would you give the card to? - What if a patient came to you with one? |
| What do you see as the benefits of using this card? | - To the patient? - To Asthma educators? - To others? |
| What do you see as the main barriers to using the card? | - Patient barriers? - Practitioner barriers? |
| What improvements could be made to the card? | - Other information? - Electronic version? |

**G. Conclusion**

For the last five minutes of today’s focus group, we would like you to provide a written summary of your opinions.

Could you please identify **in order of priority** what **you** consider to be important, with regard to today’s discussion about the Asthma Action Plan card?

**We thank you kindly for your time, please help yourself to any food or beverages.**

**We would also like to offer a token of our appreciation for your participation.**
