# Supplementary material for: A qualitative evaluation of the implementation of guidelines and a support tool for asthma management in primary care
Source: Asthma Res Pract. 2016 May 4;2:8. doi: 10.1186/s40733-016-0023-9 (PMC5142429; doi:10.1186/s40733-016-0023-9)
Supplement: Additional file 2: — Patients with asthma at risk: Tools implementation evaluation study. (PDF 84 kb) [file 40733_2016_23_MOESM2_ESM.pdf]

PATIENTS WITH ASTHMA AT RISK: TOOLS IMPLEMENTATION EVALUATION STUDY (PARTIES)

FOCUS GROUP DISCUSSION (Pharmacists)

SUMMARY SHEET

Please identify **in order of priority** what is important to you, considering today's discussion, regarding the Asthma Action Plan card.

1. Reasons to use the Asthma Action Plan card?

   a. ____

   b. ____

   c. ____

2. Reasons **NOT** to use the Asthma Action Plan card?

   a. ____

   b. ____

   c. ____

3. Any other comments?

   ____

   ____

   ____

   ____

   ____
